# Mirvetuximab soravtansine: A breakthrough in targeted therapy for platinum-resistant ovarian cancer

**DOI:** 10.1097/MD.0000000000038132

**Published:** 2024-05-17

**Authors:** Emmanuel Kokori, Gbolahan Olatunji, Rosemary Komolafe, Israel Charles Abraham, Bonaventure Ukoaka, Owolabi Samuel, Akinmeji Ayodeji, Ibukunoluwa Ogunbowale, Chidiogo Ezenwoba, Nicholas Aderinto

**Affiliations:** a Department of Medicine and Surgery, University of Ilorin, Ilorin, Nigeria; b Department of Internal Medicine, Asokoro District Hospital, Abuja, Nigeria; c Department of Medicine and Surgery, Lagos State Health Service Commission, Lagos, Nigeria; d Department of Medicine and Surgery, Olabisi Onabanjo University, Ogun, Nigeria; e Department of Medicine and Surgery, Babcock University Teaching Hospital, Ogun, Nigeria; f Department of Medicine and Surgery, Afe Babalola University, Ado-ekiti, Ekiti, Nigeria; g Department of Medicine and Surgery, Ladoke Akintola University of Technology, Ogbomoso, Nigeria.

**Keywords:** folate receptor alpha (FRα), mirvetuximab soravtansine, ovarian cancer, platinum-resistant

## Abstract

Ovarian cancer, ranked as the second leading cause of gynecologic malignancy-related deaths globally, poses a formidable challenge despite advances in early detection and treatment modalities. This paper explores the efficacy and safety of mirvetuximab soravtansine, the first folate receptor alpha (FRα)-targeting antibody-drug conjugate, in platinum-resistant ovarian cancer expressing FRα. A review of 4 key studies involving 453 participants consistently demonstrates mirvetuximab soravtansine’s clinically meaningful antitumor activity and favorable safety profile. Clinical implications emphasize mirvetuximab soravtansine’s pivotal role in targeted therapy, especially for high FRα-expressing tumors, potentially reshaping platinum-resistant ovarian cancer management. The combination therapy approach introduces a novel dimension, suggesting enhanced therapeutic outcomes. Even in heavily pretreated patients, mirvetuximab soravtansine’s favorable tolerability positions it as a viable option. The reliability of archival tissue for FRα assessment simplifies patient selection, streamlining accessibility to targeted therapies. However, identified gaps, including limited diversity in patient populations, sparse quality of life data, and the need for long-term safety information, indicate areas for future research. Exploration of additional biomarkers predicting mirvetuximab soravtansine responsiveness is essential for personalized treatment.

## 1. Introduction

In 2020, ovarian cancer, with 22,000 annual diagnoses and an estimated 207,252 deaths, ranked as the second leading cause of death from gynecologic malignancies globally.^[1]^ This cancer, the eleventh most common cancer among females, represents the fifth leading cause of cancer-related death in women.^[1,2]^ Despite advancements in early detection strategies, such as comprehensive evaluations involving gynecology specialist reviews, Transvaginal ultrasound, and CA-125 assays, the morbidity and mortality of ovarian cancer remain largely unaffected.^[3]^ Current treatment modalities, including surgery and platinum-based chemotherapy, supplemented with antiangiogenic bevacizumab and poly(ADP-ribose) polymerase inhibitors, have gained prominence in the past decade.^[3]^ However, the challenge persists as the majority of patients diagnosed with advanced-stage disease experience recurrence despite initial treatment.^[4]^

Platinum-based chemotherapy has traditionally been the mainstay of treatment for ovarian cancer.^[5]^ However, a subset of patients develops resistance to platinum-based therapies, presenting a formidable challenge in the management of the disease.^[6]^ This platinum-resistant phenotype necessitates exploring alternative therapeutic strategies to improve outcomes for affected individuals.^[7]^ Folate receptor alpha (FRα), a glycoprotein overexpressed in several epithelial malignancies, including ovarian cancer, has emerged as a promising target for biological therapy.^[8]^ With approximately 80% of epithelial ovarian cancer tumors constitutively expressing FRα, the receptor’s selective presence offers a targeted approach to treatment.^[7,8]^

Folate receptors, especially FRα, exhibit differential expression, making them attractive for antibody-drug conjugate (ADC)-based strategies.^[9]^ Mirvetuximab soravtansine, the first FRα-targeting ADC, involves a rational design with a humanized FRα binding monoclonal antibody linked to the cytotoxic maytansinoid derivative.^[10]^ The ADC’s high-affinity binding, followed by internalization, results in intracellular accumulation of maytansinoid derivative, disrupting the microtubule network, inducing cell cycle arrest, and leading to apoptotic cell death.^[11]^ Additionally, the catabolites may induce bystander killing, making mirvetuximab soravtansine (MIRV) active against tumors with heterogeneous FRα expression.^[12–14]^ Clinical trials with single-agent mirvetuximab soravtansine have demonstrated encouraging anticancer activity and a tolerable safety profile. The paper evaluates the efficacy and safety of mirvetuximab soravtansine in patients with platinum-resistant ovarian cancer expressing FRα.

## 2. Methodology

A literature search was conducted to identify relevant studies. PubMed, Embase, and Cochrane Library databases were searched for articles published up to January 2024. The following keywords and their combinations were used: “Mirvetuximab soravtansine,” “FRα-positive ovarian cancer,” “platinum-resistant ovarian cancer,” and related terms. Additional filters were applied to focus on human studies and articles in English. Inclusion criteria encompassed studies evaluating mirvetuximab soravtansine in the context of FRα-positive platinum-resistant ovarian cancer. Studies involving clinical trials and observational studies were considered. Relevant data were extracted from selected studies, including study design, patient characteristics, intervention details, outcome measures, and key findings. A narrative synthesis approach was employed to integrate and interpret findings from diverse studies. Themes and patterns across studies were identified, and results were organized to provide a comprehensive overview of the knowledge of mirvetuximab soravtansine in FRα-positive platinum-resistant ovarian cancer.

## 3. Review of current evidence

There are currently 4 studies (Matulonis et al^[15]^, Martin et al^[8]^, Moore et al^[16]^, Gilbert et al^[17]^) of focus on the efficacy and safety of mirvetuximab soravtansine in patients with platinum-resistant ovarian cancer expressing FRα (Table 1). These studies collectively involved a total of 453 participants with platinum-resistant ovarian cancer. Mirvetuximab soravtansine was administered at a consistent dose of 6 mg/kg, with administration intervals ranging from once every 3 weeks to specific study protocols. The median duration of response varied across studies, with notable efficacy observed over 6.9 to 9.7 months. The overall objective response rate (ORR) across the studies ranged from 22% to 44%. Moreover, mirvetuximab soravtansine consistently demonstrated clinically meaningful antitumor activity, with encouraging response rates observed in patients with 1 to 2 prior therapies (35.3%) and those with 3 prior therapies (30.2%). Common treatment-related adverse events included blurred vision, keratopathy, and nausea. Despite these, mirvetuximab soravtansine displayed a favorable safety profile, with lower rates of grade 3 or higher adverse events than standard chemotherapy.

**Table 1:** Current studies.

Author (study name) & year	Study design	Patient population and study size	Intervention	Efficacy outcome	Safety outcome	Other key outcomes
Matulonis et al (SORAYA) 2023^[15]^	Single arm, phase II, international study	106 patients, who were 18 years old or older, with platinum-resistant ovarian cancer and had received 1 to 3 previous treatments with other chemotherapeutic agents. It was a compulsory requirement for all patients to have received bevacizumab	Once every 3 weeks, until evidence of worsening conditions, intolerable toxicity levels, withdrawal of consent or death, patients received 6mg/kg of Mirvetuximab Soravtansine (adjusted for body weight) once every 3 weeks, intravenously. From 1 day prior to day 8 of each cycle, patients compulsorily had to use corticosteroid eye drops and artificial lubricating tears. Before each dose and upon report of ocular symptoms, patients underwent ophthalmic examination which continued for some, until the toxicity resolved.	In 71.4% of patients in the evaluable population, there was a decrease in tumor size, with complete remission occurring in 5 patients and partial remission in 29 patients. An objective response rate of 32.4% (95% CI, 23.6 to 42.2; *P* < .0001) based on investigator assessment was recorded. The blinded independent central review efficacy evaluable population had an observed response rate of 30.2% (95% CI, 21.3 to 40.4), and complete remission occurred in 6 patients with partial remission in 23 patients.	Mirvetuximab Soravtansine showed acceptable tolerability and safety in the patient population. Adverse events related to the drug occurred in 86% of patients with at least 1 grade 3 event occurring in 28% of the patient population and reports of a grade 4 event were seen in 1% of the population. The most common adverse events were blurred vision, keratopathy and nausea. 12 out of 55 patients that had blurred vision and keratopathy required a decrease in the administered dose and one patient discontinued therapy as a result. At the point of data cutoff, no patient had permanent ocular sequalae. In total, adverse events led to a dosage delay and dosage decrease in 33% and 20% of the safety population respectively. There were 7 deaths recorded during the study, with 4 as a result of disease progression, 2 as a result of adverse events not related to the drug and one as a result of cancer metastasis to the lung. The commonest hematologic adverse events related to the drug included neutropenia, thrombocytopenia and anemia occurring in 13%, 9% and 8% of the total number of patients respectively.	Based on investigator assessment, the median duration of response was 6.9 (95% CI, 5.6 to 9.7) months and the median time to response was 1.5 (range, 1.0–5.6 months). The blinded independent central review observed a median time to response of 1.4 months (range, 1.0–5.4 months).
Martin et al 2017^[8]^	A Phase 1 expansion study	27 patients who were at least 18 years or older and had relapsed epithelial ovarian cancer after previous chemotherapy with evidence of sufficient folate receptor alpha presence in the tumor using immunohistochemistry.	Patients were treated with 6.0mg/kg (adjusted for ideal body weight) of intravenous mirvetuximab soravtansine until unacceptable toxicity levels, worsening disease conditions, or patient-based or investigator-based decisions. Patients were grouped based on membranous folate receptor alpha staining intensity into high, medium, and low levels of folate receptor alpha demonstration.	The objective response rate was 22% with complete remission seen in 2 patients and partial remission seen in 4 patients. Based on archival folate receptor alpha positivity expression, the objective response rate was 31%, 20%, and lack of objective response in the high, medium, and low subgroups respectively. Thus, the clinical efficacy of mirvetuximab sorvatansine was observed to be dependent on folate receptor alpha expression.	26 patients reported drug related adverse events (all of which were either grade 1 or 2) with the most common being keratopathy, fatigue, diarrhea and blurred vision in 48%, 44%, 37%, and 37% in the patient population respectively. There were no deaths related to the treatment, corneal anomalies and blurred vision were minor and rescindable. Hypokalemia and organizing pneumonia (both with grades of at least 3 or more) were seen in 2 patients which resolved after stoppage and retirement from the trial.	After 2 doses of chemotherapy, folate receptor alpha levels remained stable in most of the patients however, some declines were recorded. There was also a 71% concordance rate between archival tumor biopsy samples and pretreatment biopsy samples in the levels of folate receptor alpha in eligible patients.
Moore et al (MIRASOL) 2023^[16]^	A controlled, randomized, open-label, confirmatory, international study	453 patients, each of them being at least 18 years old, with a confirmed diagnosis of platinum resistant ovarian cancer, having received 1 to 3 past lines of chemotherapy, and experienced disease progression in spite of treatment. Patients were randomized into 2 groups: 227 patients in the mirvetuximab soravtansine subgroup and 226 patients in the chemotherapy group.	At the investigator’s discretion, patients in the mirvetuximab soravtansine group received paracetamol, dexamethasone, and diphenhydramine for injection reactions before receiving intravenous mirvetuximab soravtansine at 6mg/kg of adjusted ideal body weight once in 3 weeks until consent withdrawal, intolerable toxicity effects, worsening disease conditions or death. Glucocorticoid eyedrops were also given prophylactically 6 times a day from the day before the administration of mirvetuximab soravtansine to day 4, and 4 times daily from days 5 to 8 per cycle. Patients in the chemotherapy subgroup received one of 3 drugs determined by the investigators; 92 were given intravenous paclitaxel at a dosage of 80mg/m^2^ of body surface area on the 1st, 8th,15th and 22nd days of a 4 week cycle, 81 were given intravenous pegylated liposomal doxorubicin at a dosage of 40mg/m^2^ on the 1st day of a 4 week cycle, 53 were given intravenous topotecan at a dosage of 4mg/m^2^ on the 1st, 8th, and 15th days of a 4 week cycle or 1.25mg/m^2^ on from the 1st through the 5th days of 1 3 week cycle.	Mivetuximab soravtansine demonstrated superior results compared to chemotherapy after investigators had assessed progression free survival, objective response, and overall survival in the 2 randomized groups. Median progression free survival was 5.62 (95% CI, 4.34 to 5.95) months in the mirvetuximab soravtansine subgroup and 3.98 (95% CI, 2.86 to 4.47) months in the chemotherapy subgroup. Objective response rate in the mirvetuximab soravtansine group was 42.3 percent (95% CI, 35.8 to 49.0) and 15.9 percent (95% CI, 11.4 to 21.4) in the chemotherapy subgroup (odds ratio, 3.81; 95% CI, 2.44 to 5.94; *P* < .001). Complete remission and partial remission were seen in 5.3% (12) and 37.0% (84) of patients in the mirvetuximab soravtansin subgroup respectively compared to 0 (0) percent complte remission and 15.9 (36) percent partial remission in the chemotherapy subgroup. The mirvetuximab soravtansine subgroup had a median overall survival of 16.46 months (95% CI, 14.46 to 24.57) compared to 12.75 months (95% CI, 10.91 to 14.36) recorded in the chemotherapy subgroup. This was significant in terms of the hazard ratio for death which showed that a patient on mirvetuximab soravtansine was less likely to die than a patient on chemotherapy (hazard ratio for death, 0.67; 95% CI, 0.50 to 0.89; *P* = .005).	210 participants in the mirvetuximab soravtansine subgroup reported adverse events versus 194 in the chemotherapy group. Commonly occurring adverse events in the mirvetuximab soravtansine subgroup were blurred vision (40.8%), keratopathy (32.1%), abdominal pain (30.3%) and fatigue (30.3%) with anemia (34.3%), nausea (29.0%), neutropenia (28.5%), and fatigue (25.1%) being the most common adverse events in the chemotherapy subgroup. The commonest adverse events leading to stoppage of mirvetuximab sorvantansine medication were blurred vision and pnneumonitis occurring in 3 patients each, with 20 patients in total discontinuing mirvetuximab soravtansine due to adverse events. 33 patients stopped chemotherapy due to adverse events with the commonest being peripheral neuropathy (in 4 patients), thrombocytopenia (in 3 patients), and fatigue in 3 patients. 1 patient died of neutropenic sepsis in the mirvetuximab soravtansine subgroup and 1 patient died of septic shock in the chemotherapy subgroup and both deaths were drug related.	Amongst the 96 patients in the mirvetuximab soravtansine subgroup that showed a response, the median duration of response was 6.77 months (95% CI, 5.62 to 8.31) and 4.47 months (95% CI, 4.17 to 5.82) amongst the 36 participants in the chemotherapy subgroup that showed a response. There was a difference of 27.7 (95% CI, 17.5 to 37.9) percentage points between both groups in the percentage of participants that showed a CA-125 response with the mirvetuximab soravtansine group having a higher percentage (58.0 s 30.3).
Gilbert et al () 2023^[17]^	A phase 1b/2, multi-arm trial	94 patients that were at least 18 years old, with platinum resistant epithelial ovarian cancer (77%), fallopian tube cancer (18%) or primary peritoneal cancer (5%) confirmed by histology, who had received a minimum of 1 previous systemic chemotherapy treatment and a maximum of 3 previous systemic chemotherapy treatment.	In accordance with adjusted ideal body weight, patients received mirvetuximab soravtansine at a dose of 6mg/kg via the intravenous route, after which they received 15mg/kg of bevacizumab on the first day of a 21-day treatment cycle. Corticosteroid eye drops were administered prophylactically from day 1 to day 8 of the treatment cycle as well as daily administration of lubricating artificial tears free of preservatives in order to cater for anticipated ocular symptoms. If patients stopped using either of bevacizumab or mirvetuximab sovantansine, they were allowed to continue single therapy with the other drug. Treatments continued until there was either consent withdrawal, worsening disease conditions, or unacceptable levels of toxicity.	The drug combination showed higher efficacy in tumors with higher expression of folate receptor alpha. The objective response rate was 44% (95% CI, 33, 54) with complete and partial remission seen in 5 and 36 patients respectively. 9.7 months (95% CI, 6.9, 14.1) and 8.2 months (95% CI, 6.8, 10.0) were the median duration of response and median progression free survival respectively.	93 patients experienced at least one drug-related adverse event with the commonest events of any grade being blured vision (57%), diarrhea (54%), nausea (51%), and fatigue (43%). Most of the adverse events were mild and patients did not report any long-term sequelae. 36 patients experienced serious adverse events with the most common being small intestinal obstruction, diarrhea, and gastrointestinal hemorrhage. Drug related adverse events led to drug discontinuation in 30 patients with the most common being thrombocytopenia (7%), peripheral neuropathy (3%), gastrointestinal hemorrhage (3%), and pneumonitis (2%). In the single patient who died during the study, the death was deemed to be related to bevacizumab (intestinal perforation).	Patients who had never received bevacizumab as part of previous therapy showed better response to the drug combination compared to those who had previous bevacizumab therapy. In the bevacizumab unexposed group, the objective response rate was 56%, median duration of response was 10.4 months and median progression free survival was 10.6 months compared to an objective response rate of 35%, median duration of response of 9.7 months, and median progression free survival of 6.8 months in the bevazizumab exposed group.

CI = confidence interval, SORAYA = Single-Arm, Phase II Study of Mirvetuximab Soravtansine.

The Single-Arm, Phase II Study of mirvetuximab soravtansine study, a phase II single-arm trial, included 106 FRα-high patients with platinum-resistant epithelial ovarian cancer who had received 1 to 3 prior therapies, including bevacizumab.^[15]^ The primary endpoint, confirmed ORR by the investigator, was 32.4%, with a median duration of response of 6.9 months. Notably, patients with 1 to 2 prior lines of therapy exhibited an ORR of 35.3%, emphasizing MIRV’s effectiveness in this subgroup. Concordance between archival and biopsy samples in the phase I expansion study by Martin et al^[8]^ supported the reliable identification of FRα-positive tumors using archival tissue, making it suitable for patient selection in MIRV clinical trials. The study observed an ORR of 22%, with superior efficacy in patients exhibiting the highest FRα levels. Adverse events were generally mild, with keratopathy, fatigue, diarrhea, and blurred vision is the most common.

The phase 3 trial comparing mirvetuximab soravtansine with chemotherapy in platinum-resistant, high-grade serous ovarian cancer revealed significant benefits with mirvetuximab soravtansine.^[16]^ The mirvetuximab soravtansine group showed a median progression-free survival of 5.62 months compared to 3.98 months with chemotherapy. An impressive ORR of 42.3% was observed in the MIRV group, along with longer overall survival (16.46 months vs 12.75 months). Fewer grade 3 or higher adverse events occurred with mirvetuximab soravtansine, emphasizing its favorable safety profile. Furthermore, the combination of mirvetuximab soravtansine and bevacizumab demonstrated promising results in patients with recurrent platinum-resistant ovarian cancer, with an impressive 44% confirmed ORR, including complete responses.^[17]^ The median duration of response was 9.7 months, and the median progression-free survival was 8.2 months. The combination therapy was well-tolerated, with blurred vision, diarrhea, and nausea being the most common adverse events.

## 4. Clinical implications and future directions

The findings across the studies collectively suggest significant clinical implications for using mirvetuximab soravtansine in treating platinum-resistant ovarian cancer with high FRα expression. Consistent efficacy in patients with high FRα expression signifies a pivotal advancement in targeted therapies. Mirvetuximab soravtansine’s ability to specifically target FRα-positive tumors establishes it as a promising treatment option for a biomarker-selected population, presenting a potential paradigm shift in managing platinum-resistant ovarian cancer.^[18]^ In addition, exploring mirvetuximab soravtansine in combination with bevacizumab introduces a compelling dimension to treatment strategies. The promising results from this combination therapy suggest that a dual approach, leveraging the unique mechanisms of both agents, holds the potential for enhanced therapeutic outcomes in platinum-resistant ovarian cancer.^[19]^ This avenue warrants further exploration and may open new avenues for combinatorial approaches in the field.

One of the standout features of mirvetuximab soravtansine is its favorable tolerability and safety profile, even in patients with a history of heavy pretreatment. This characteristic positions mirvetuximab soravtansine as a viable and well-tolerated option, particularly for individuals who may have exhausted other treatment alternatives.^[20]^ The ability to maintain a positive safety profile while providing meaningful therapeutic effects is a crucial aspect in the context of managing advanced ovarian cancer.^[21]^ Similarly, Martin et al (2017) study contributes a valuable perspective by emphasizing the reliability of archival tissue for identifying FRα-positive tumors. This finding simplifies patient selection in clinical trials and, potentially, routine clinical practice. The validation of archival tissue as a dependable source for biomarker assessment reduces procedural complexities and facilitates broader accessibility to targeted therapies like mirvetuximab soravtansine, thereby streamlining the patient identification process.^[22]^

One notable gap across the studies lies in the predominant inclusion of patients with high-grade serous ovarian cancer. This specific focus limits the generalizability of findings to other histological subtypes. A more diverse representation of ovarian cancer subtypes within the study populations is essential for a comprehensive understanding of mirvetuximab soravtansine’s efficacy across the spectrum of the disease. Also, an important aspect overlooked in the current body of evidence is the limited exploration of the impact of mirvetuximab soravtansine treatment on patients’ quality of life. Chronic diseases, such as ovarian cancer, necessitate a holistic approach that considers not only therapeutic efficacy but also the overall well-being of patients.^[23]^ Future studies should incorporate comprehensive assessments of quality of life parameters to provide a more nuanced perspective on the treatment’s impact beyond clinical endpoints. While the available studies offer insights into the short-term safety profile of mirvetuximab soravtansine, a notable gap exists concerning long-term safety data. Prolonged use of therapeutic agents raises concerns about cumulative toxicities and late-emerging adverse effects. To address this gap, future studies should prioritize collecting and analyzing long-term safety data, ensuring a more thorough understanding of the treatment’s safety profile over extended periods. Moreover, the studies recognize the significance of FRα expression as a key biomarker, there is a gap in exploring other potential biomarkers that could predict response to mirvetuximab soravtansine. Identifying additional biomarkers may enhance the precision of patient selection, allowing for a more tailored and effective therapeutic approach. Future investigations should delve into discovering and validating supplementary biomarkers that contribute to a comprehensive predictive model for mirvetuximab soravtansine responsiveness.

## 5. Limitations of study

The predominant focus on high-grade serous ovarian cancer in the reviewed studies limits the generalizability of findings to other histological subtypes. A more diverse representation of ovarian cancer subtypes within the study populations is necessary for a comprehensive understanding of mirvetuximab soravtansine’s efficacy across the spectrum of the disease. In addition, while the available studies offer insights into the short-term safety profile of mirvetuximab soravtansine, a notable gap exists concerning long-term safety data. Prolonged use of therapeutic agents raises concerns about cumulative toxicities and late-emerging adverse effects. Future studies should prioritize collecting and analyzing long-term safety data to ensure a more thorough understanding of the treatment’s safety profile over extended periods.

## 6. Conclusion

This paper synthesizes the current evidence on the efficacy and safety of mirvetuximab soravtansine in the context of platinum-resistant ovarian cancer with high FRα expression. The studies examined collectively demonstrate consistent and clinically meaningful antitumor activity of mirvetuximab soravtansine, establishing it as a promising therapeutic option for this biomarker-selected population. The findings highlight the efficacy of mirvetuximab soravtansine in patients with high FRα expression, underscoring its potential as a targeted therapy for this specific subgroup. Moreover, the exploration of mirvetuximab soravtansine in combination with bevacizumab introduces a promising avenue for enhancing therapeutic outcomes in platinum-resistant ovarian cancer. The dual approach exhibits encouraging results, suggesting that leveraging the unique mechanisms of both agents may provide a synergistic effect. However, identified gaps in the studies present opportunities for future research. The predominant focus on high-grade serous ovarian cancer limits the generalizability of findings to other histological subtypes. Future studies should strive for a more diverse representation of ovarian cancer subtypes to ensure a comprehensive understanding of MIRV’s efficacy across the disease spectrum. Furthermore, the limited exploration of the impact on patients’ quality of life and the absence of long-term safety data are critical gaps that warrant attention in future studies.

## Author contributions

**Conceptualization:** Emmanuel Kokori.

**Writing – original draft:** Emmanuel Kokori, Gbolahan Olatunji, Rosemary Komolafe, Israel Charles Abraham, Bonaventure Ukoaka, Owolabi Samuel, Akinmeji Ayodeji, Ibukunoluwa Ogunbowale, Chidiogo Ezenwoba, Nicholas Aderinto.

**Writing – review & editing:** Emmanuel Kokori, Gbolahan Olatunji, Rosemary Komolafe, Israel Charles Abraham, Bonaventure Ukoaka, Owolabi Samuel, Akinmeji Ayodeji, Ibukunoluwa Ogunbowale, Chidiogo Ezenwoba, Nicholas Aderinto.
